# What Influences the Prevalence and Intensity of Haemoparasites and Ectoparasites in an Insular Lizard?

**DOI:** 10.3390/ani13040723

**Published:** 2023-02-17

**Authors:** A. Isabel Ferreira, Isabel Damas-Moreira, Kate L. A. Marshall, Ana Perera, D. James Harris

**Affiliations:** 1 BIOPOLIS Program, CIBIO-InBIO, Universidade do Porto, 4485-661 Vila do Conde, Portugal; 2 Departamento de Biologia, Universidade do Porto, 4169-007 Porto, Portugal; 3 Department of Behavioural Ecology, Bielefeld University, 33615 Bielefeld, Germany; 4 Department of Zoology, University of Cambridge, Cambridge CB2 1TN, UK

**Keywords:** host-parasite interactions, 18S rRNA, *Podarcis erhardii*, *Hepatozoon*, *Schellackia*, *Karyolysus*, ticks, mites

## Abstract

**Simple Summary:**

Islands have long been acknowledged as model systems for studying evolution, and within these, lizards and their blood parasites can be an ideal framework to determine how island characteristics, such as size and isolation, correlate with parasite prevalence and intensity. In this study, we assessed haemogregarine parasite diversity within the Aegean wall lizard *Podarcis erhardii* across the Cyclades islands, using an integrated approach of assessing both blood slides through microscopy and genetic variability using molecular tools. We also recorded the prevalence of ectoparasites, ticks and mites. We identified two unrelated groups of blood parasites, with a species of the genus *Schellackia* being reported for the first time in this host species. The presence of ticks was associated with haemogregarine prevalence. However, in contrast to an earlier study, we did not find a significant impact of the island age and area on parasite prevalence. Despite the relative simplicity of island systems, untangling the factors underpinning parasite prevalence remains a complex task, requiring integrated approaches.

**Abstract:**

Island biogeography theories predict that characteristics such as island size, age, and isolation interplay in host-parasite dynamics. In this study, we analyzed haemogregarines of the Aegean wall lizard, *Podarcis erhardii*, to investigate how island characteristics relate to parasite prevalence and intensity. A previous assessment of 19 Greek island populations suggested that isolation time and host population density were key predictors of haemogregarines. Here, by combining microscopy and genetic techniques, we extend this previous study to four additional islands: Syros, Folegandros, Santorini and Nea Kameni. We also recorded the prevalence of ticks and mites, definitive hosts for these parasites. The genetically identified haemogregarines are part of a clade with parasites from other lizard species, including some considered as *Karyolysus*, but others assigned to *Hepatozoon*. The prevalence of these parasites differed significantly between islands, while their intensity did not. The presence of ticks was associated with endoparasite prevalence, and males were more frequently infected by haemogregarines than females. Combining our data with that of the previous study, we found no significant impact of the island age and area on parasite prevalence. We also confirmed the presence of the unrelated parasite genus *Schellackia* through microscopy and DNA sequencing, which is the first record of this genus in this host species. Our results further highlight the complexity of host-parasite systems.

## 1. Introduction

Parasites represent a huge proportion of biodiversity, and play an important role in ecosystems, influencing the dynamics and structure of host populations [1,2,3]. Islands are classic models in evolutionary studies due to the simplicity of the system when compared to continental regions [4]. It is expected that island size, age, and isolation all interplay to impact host-parasite dynamics, but the importance of the different factors is still unclear [4,5,6].

In islands, animal populations in the initial stages of colonization exhibit higher levels of inbreeding and lower genetic variability [7]. During the process of isolation, their parasites can decrease in numbers or even be entirely lost if there is a reduction in the host abundance and dispersal, or if there are “bottlenecks”, associated with each colonization event. Remarkably, parasites can also shift to other hosts [8]. On the other hand, due to the lack or reduced number of predators, insular systems can have very dense host populations, particularly on smaller islands [9]. Given this, insular host species can be more susceptible to diseases and parasitism [10].

Lizards can be an ideal system to study the variation of parasitism on island populations given their low mobility, high densities, and ease of sampling. This is evident for the Aegean Wall Lizard, *Podarcis erhardii*, that can be found in very different types of islands and habitats [11]. They are widely distributed across the Greek mainland and Aegean islands [11]. *Podarcis erhardii* is a diurnal medium-sized lizard, primarily insectivorous [12], although its diet varies considerably according to habitat [13,14]. Haemogregarines are the most common blood parasites infecting reptiles. Recently, Fornberg and Semegen [4] carried out an extensive assessment of parasite diversity within *P. erhardii* across 19 of the Cyclades islands, which are part of the Aegean archipelago. The authors analyzed the parasite prevalence (number of infected individuals in the population) and intensity (number of haemogregarines per individual) of haemogregarines for each island. They found that islands with a greater host density and islands that had been isolated for less time generally had higher haemogregarine prevalence and intensity, which they hypothesized was associated with insular density compensation (smaller islands contain denser host populations). The time when islands were spatially isolated also showed a trend towards higher prevalence and parasitaemia levels. However, in their study, the parasites were identified through microscopy, so it was not possible to ascertain whether genetically distinct parasites were present, nor to place them within a phylogenetic framework.

Two haemogregarine genera are widely reported from reptiles, *Hepatozoon* and *Karyolyus*, both from the apicomplexan order Adeleorina. *Hepatozoon* parasites are present in almost every group of terrestrial vertebrates, being one of the most abundant and widespread hemoparasites, particularly in reptiles, while *Karyolysus* are predominantly found in lacertid lizards [15]. Genetic analyses indicate that the *Karyolysus* species form a lineage within a paraphyletic Hepatozoon [16]. The lifecycle of the *Hepatozoon* species is heteroxenous, employing intermediate hosts—vertebrates– and definitive hosts—invertebrates– typically mites and ticks [17,18,19]. In the Greek islands, the definitive hosts are likely ticks (the genera *Dermacentor, Hyalomma*, and *Ixodes* have been reported from these islands); [20] or mites from the family Trombiculidae [21]. Ectoparasite load is generally affected by the type of vegetation present and the presence of ruminants [20].

**Figure 1:**
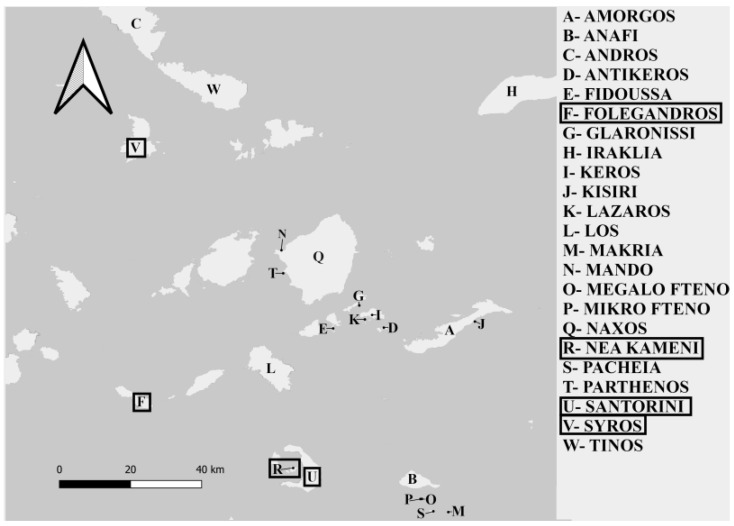
Map of the Greek islands sampled in Fornberg and Semegen (2021) and in our study. The islands included in our study were Folegandros, Syros, Santorini and Nea Kameni, and are highlighted in rectangles.

We aim to extend the work of Fornberg and Semegen [4] by analyzing the parasite prevalence and intensity in four additional islands within the Cyclades: Folegandros, Syros, Santorini and Nea Kameni (Figure 1). These islands have notable differences in size (from 3.4 km^2^ to 101.9 km^2^), time since separation (between 400 years and 12,800 years) [13,22], diversity of avian predators and anthropogenic disturbance (Table 1) [23,24]. We conducted genetic analyses by sequencing part of the 18S rRNA gene to further identify the blood parasites present. The numbers of ectoparasites attached to the lizards were also recorded in order to determine whether these are associated with the presence of haemogregarines.

## 2. Materials and Methods

Blood samples from 195 adult *P. erhardii* were collected between April and June 2014 across the 4 islands (08.4 to 24.4 Syros, 30.4 to 12.5 Folegandros, 14.5 to 01.6 Santorini, 27.5 to 02.6 Nea Kameni). After the lizards were caught, the tail tip was cut, and blood smears were performed from the resulting bleeding. The tip of the tail was stored in 96% ethanol. Clipping material in this way is a common technique in herpetological studies and has been shown to not induce significant increases in corticosterone levels, indicating this generates relatively little stress [25]. The lizards were sexed, and the snout-vent length (SVL) and weight were recorded using digital callipers (±0.01 mm) and a small scale (±0.01 g), respectively. An estimation of the number of ticks and mites present in each individual was also recorded by the same researcher to minimize errors. The lizards were then released in the same place they were captured. The blood smears were air dried and fixed with methanol on the same day. Once in the laboratory, they were stained with Giemsa for 45 min.

The blood smears were examined under an Olympus CX41 microscope using ×400 optics, and pictures were taken with Cell^B 3.4 Olympus^®^ software (Olympus, Münster, Germany). These pictures were inspected using the ImageJ 1.46^®^ program [26]. For each individual, we counted 2000 erythrocytes, and scored how many were infected with parasites. We considered the prevalence as an estimate of the number of infected individuals in the sampled population and the intensity as the number of parasitized erythrocytes per individual.

All of the statistical analyses were performed in R v4.0.4 [27]. We employed generalized linear models with a binomial distribution to determine whether the island and host characteristics (sex, body size, and weight) had an effect on the prevalence of hemoparasites and ectoparasites. For analyzing the parasite intensity, we employed permutational analysis of the variance using the function *adonis* [28], in the package *vegan* (Permanova, [29]. Finally, we combined our data with the data from Fornberg and Semegen [4] using a Permanova analysis to determine whether the island age and area influenced the prevalence and intensity of endoparasites.

To ascertain the genetic identity of the parasites, we extracted DNA from six tail-tip tissues using the standard High Salt methods [30], and then performed a PCR to amplify part of the 18s rRNA gene, using the Hep300 and Hep900 primers from Ujvari [31] and the conditions described by Maia [32]. In brief, the PCR were performed in a 15 μL final volume, consisting of PCR buffer at 1 × concentration, MgCl_2_ at 1.5 mM, dNTPs at a concentration of 0.2 mM for each nucleotide, each primer at 0.5 μM, Taq polymerase at 0.025 units/μL, and 1 μL of extracted DNA. The PCR reaction consisted of 35 iterations of the following cycle: 30 s. at 94 °C, 30 s. at 60 °C and 1 min. at 72 °C, beginning with an additional denaturation step of 3 min. at 94 °C and ending with a final extension at 72 °C for 10 min. Positive PCR products were cleaned and sequenced by a commercial company (Genewiz, Germany). All of the new sequences were submitted to GenBank (Accession numbers OQ415536 to OQ415540). The sequences were aligned in Geneious Prime 2021.1.1 (Biomatters Ltd, Auckland, New Zealand) using clustalW, with representative sequences from the same genus present in GenBank (104 sequences). We used Bayesian inference to estimate the phylogeny with the most appropriate model of molecular evolution identified using PartitionFinder2 [33]. Bayesian inference was implemented using Mr. Bayes v.3.2.7 [34]. The analysis was run for ten million generations, saving one tree every 1000 generations. The log-likelihood values of the sample points were plotted against the generation time and all the trees prior to reaching stationarity (25%) were discarded. The remaining trees were combined in a 50% majority-rule consensus tree [34].

## 3. Results

The lizards’ body sizes differed between islands (F3,191 = 23.78, *p* < 0.001) and sexes, with males being larger than females (F3,191 = 21.81, *p* < 0.001). The interaction between island and sex was not significant (F3,191 = 1.30, *p* = 0.285). The largest lizards were found in Syros, followed by Folegandros and Santorini, and finally Nea Kameni.

The haemogregarine prevalence varied between islands (Deviance = 230.81, df = 3, *p* < 0.001) and sexes (Deviance = 224.56, df = 1, *p* < 0.05), with more infected males than females. Between the islands, Folegandros had the highest prevalence, followed by Santorini and Syros, with the lower prevalence found on Nea Kameni. None of the variables included in the model, SVL (F2,190 = 0.15, *p* = 0.710), sex (F2,190 = 0.06, *p* = 0.060) nor island (F2,190 = 0.98, *p* = 0.981) had a significant influence on the intensity of the haemogregarines (Table 1).

**Figure 2:**
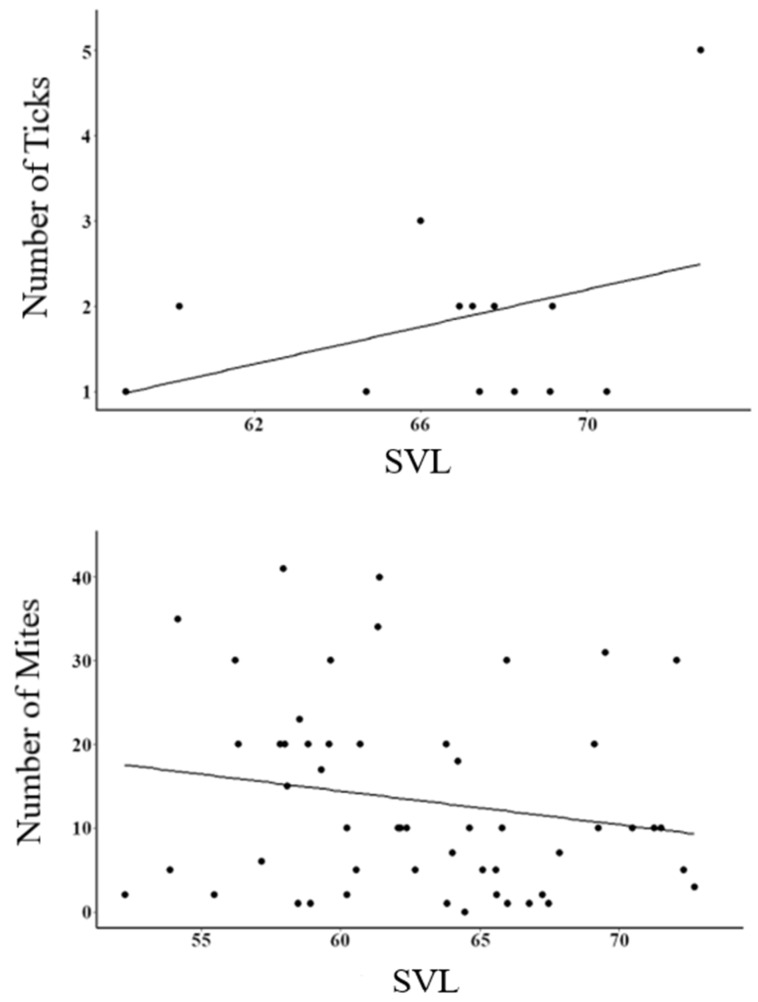
Relationship between snout-vent-length (SVL; in mm) and the number of ticks and mites found in *P. erhardii* individuals. Black lines represent linear models.

The number of ticks (F1,191 = 4.53, *p* < 0.05) and mites (F1,191 = F15.43, *p* < 0.05) found on the lizards were correlated with SVL (Figure 2), with larger individuals hosting a higher number of ticks but, inversely, a smaller number of mites. The lizards with more ticks attached had a higher probability of being infected with haemogregarines (Deviance = 2.29, df = 1, *p* < 0.05), while the number of mites and the interaction of mites and ticks did not have a significant effect on the number of haemogregarines (Deviance = 6.79, df = 1, *p* = 0.130; Deviance = 0.13, df = 1, *p* = 0.720, respectively).

Combining our results with those from Fornberg and Semegen [4], we found that the haemogregarine prevalence was not significantly influenced by either the island age (F1,21 = 1.03, *p* = 0.345), or island size (F1,21 = 0.05, *p* = 0.845), and neither was the haemogregarine intensity (islands age (F1,21 = 0.62, *p* = 0.310) and area (F1,21 = 1.03, *p* = 0.229)).

**Figure 3:**
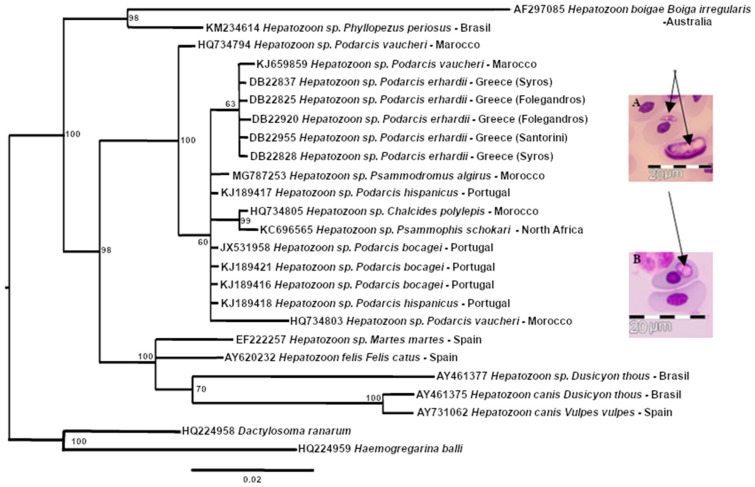
Estimate of relationships of haemogregarines based on 18S rRNA gene fragments. Numbers near to nodes represent Bayesian posterior probability values. The tree was rooted using *Dactylosoma ranarum* and *Haemogregarina balli*. Examples of endoparasites found parasitizing *Podarcis erhardii* through microscopy screening, are shown next to the phylogenetic tree: (**A**) Haemogregarines and (**B**) *Schellackia* (respective parasites indicated with arrows). Note that in (**A**) while the host nucleus is severely distorted, it is not fragmented in the manner associated with infection by *Karyolysus* sp.

The molecular analysis included five haemogregarine samples: from two lizards from Syros, two from Folegandros and one from Santorini (aligned length 553 bp). All of the new sequences were closely related (one nucleotide difference due to a heterozygotic position was found in one sample from Syros and one sample from Folegandros) and formed a clade with other haemogregarines, predominantly from lizards identified either as Karyolysus or Hepatozoon (Figure 3). Moreover, during the microscopy screening, we detected several lizards apparently infected with another unrelated endoparasite, *Schellackia* sp. (Apicomplexa, Lankesterellidae. Figure 3 and Table 1). We amplified one of these samples from Nea Kameni using the same conditions and primers as those used for the Adeleorina parasites. The sequence quality was suboptimal, but a BLAST search with 253 bp of sequence showed a 100% match with the MG775272 sequence from GenBank, from the *Schellackia* species infecting a *Timon lepidus* collected in Spain.

## 4. Discussion

Unravelling the dynamics of host-parasite relationships is a major aim in evolutionary ecology but is extremely complex, with multiple factors influencing such interactions [35]. Islands can be ideal models, simplifying the system and allowing the impacts of population fragmentation to be determined. Our results show that the haemogregarine prevalence in *P. erhardii* varies between islands and has a positive correlation with the presence and number of ticks, while the haemogregarine intensity is not affected by any of our study variables. On the other hand, ticks were only recorded on one, and mites on two, of the four islands (Folegandros, and Folegandros and Santorini, respectively), while haemogregarines were present on all four islands. Tick prevalence is associated with past grazing practices in the islands occupied by *P. erhardii* [22]. Moreover, infection with mites and ticks may change along the year, as seen in *Podarcis melisellensis*, where infections by these ectoparasites were more severe at the end of the reproductive season [36]. It seems likely that temporal variation combined with contrasting habitat characteristics and use may explain the differences in the ectoparasite prevalence in our study (Table 1), although this needs further assessment.

Unexpectedly, we found an inverse relationship between ticks and mites, with larger lizards having more ticks but fewer mites. Previous studies found that mite intensity was not associated with body size in *Podarcis muralis* [37], while in a community of three lacertid lizards in the Iberian Peninsula, there was a positive correlation between SVL and mite infestation [38]. This latter study highlighted that each host-parasite system showed unique particularities, despite involving related hosts in similar environments. An inverse relationship was also described between different habitats for both parasites, with mites being more present in areas with dry grasslands and little disturbance, and ticks in areas with higher plant cover and disturbance by livestock [39]. Our results corroborate this—even in a simplified island system, unique particularities are seen between different ectoparasites and endoparasites. Such singularities highlight the difficulties in generalizing the patterns of host-parasite dynamics.

As expected, the average sizes of *P. erhardii* vary across populations. Body size often evolves rapidly in island lizards [40]. These island populations also show colour differences that match their background colours on each island (modelled to avian predator vision), likely for camouflage [41], and the body size differences may also be associated with anti-predator defence mechanisms [42].

Recent applications of molecular tools have highlighted the diversity of the parasites infecting Mediterranean lizards. The most common parasites infecting lacertids are haemogregarines, typically considered to be transmitted by mites and ticks [20]. Less common are haemococcidians, including *Schellackia*, which are regarded as being more host-specific [43]. Our identification of the *Schellackia* species (through microscopy and molecular techniques) in all four of the studied populations is, to the best of our knowledge, the first record for *P. erhardii*. Interestingly, this parasite was not reported in the other Greek islands by Fornberg and Semegen [4], possibly due to the fact that their study did not include molecular detection. This genus of parasites generally has lower prevalence, and morphologically might be mistaken for immature gamonts of *Hepatozoon*. In our case, molecular tools were helpful to confirm its presence. The assessment of more islands for the presence *of Schellackia* would be needed to determine whether it might be more widespread but has been overlooked.

Concerning the island age and area, our data do not support the results from Fornberg and Semegen [4], where they suggested that smaller islands had a higher prevalence, despite sampling across approximately the same season (April to June in this study, May to July in Fornberg and Semegen [4]). Our results did not identify a significant relation between the island age, area and prevalence or intensity of parasitism. We suggest that island-specific characteristics, particularly the presence of domestic ruminants and associated ectoparasites, may be more important, and that this aspect deserves further investigation. Indeed, the lack of consistency between our results and the previous study by Fornberg and Semegen [4] highlights the need for more studies to understand how parasites can be shaped by the size and age of the island, vegetation, habitat structure, and lizards’ density, among other factors.

## 5. Conclusions

In contrast to the previous study of Fornberg and Semegen [4], we did not identify a significant relation between the island age, area and prevalence or intensity of parasitism. One difference between the two studies is that we identified a species of the genus *Schellackia* in all four studied populations, while Fornberg and Semegen [4] did not report any infections with this parasite. This highlights the importance of using molecular tools to confirm the diagnosis of morphologically similar, but genetically distinct, haemogregarines. In the pond turtle *Mauremys leprosa*, infection by a single blood parasite lineage showed no impact on the body condition, but infection by two lineages was associated with lower values of hematocrit [44]. The impact of these blood parasites on lizards is not fully understood, although Damas [45] found no evidence of an impact on the circulating blood cells or the hosts’ immune system. This island system may therefore be useful not only to better elucidate the dynamics of host-parasite relationships, but also to assess the conservation implications of mixed infections.

## Figures and Tables

**Table 1:** Summarized information about the islands, lizards, and parasite prevalence and intensity in this study. The description of each island includes the island traits and the number of avian predators (from the genus *Buteo*; *Falco*; *Tyto* and *Corvus*). The mean (with respective standard deviation) SVL and weight for each island population, for males (M) and females (F), and the mean parasite prevalence and intensity for each lizard population are also included.

	Island Characteristics				Lizard Characteristics				Parasite Prevalence			Parasite Intensity			
	Area (km^2^)	Number of Inhabitants	Type of Habitat	Avian Predators	Sex	SVL (mm)	Mass (g)	Haemogregarines	*Schellackia*	Ticks	Mites	Haemogregarines	*Schellackia*	Ticks	Mites
Syros	101.9	21,507	Rocky shrubland	5	35 M	69.6 ± 4.7	8.8 ± 1.7	54.3 ± 11.2	14.3 ± 1.1	-	-	6.2 ± 11.2	0.4 ± 1.1	-	-
					22 F	67.0 ± 5.5	6.6 ± 1.6	31.8 ± 14.4	4.5 ± 3.2	-	-	6.2 ± 14.4	0.7 ± 3.2	-	-
Folegandros	32.38	800	Rocky shrubland	2	37 M	67.3 ± 4.3	7.8 ± 1.5	84.2 ± 24.8	2.6 ± 0.2	50 ± 1.4	23.7 ± 3.9	9.8 ± 24.8	0 ± 0.2	0.9 ± 1.4	1.3 ± 3.9
					26 F	64.2 ± 4.7	5.5 ± 0.9	57.7 ± 10.6	7.7 ± 0.4	11.5 ± 0.6	15.4 ± 1.9	5.9 ± 10.6	0.1 ± 0.4	0.2 ± 0.6	0.5 ± 1.9
Santorini	76.19	13,500	Rocky shrubland	5	25 M	64.4 ± 4.9	7.1 ± 1.8	69.2 ± 7.7	38.5 ± 0.9	-	92.3 ± 11.5	5.1 ± 7.7	0.6 ± 0.9	-	15.1 ± 11.5
					25 F	59.4 ± 4.6	4.6 ± 1.0	64 ± 4.8	36 ± 4.8	-	88 ± 1.8	7 ± 4.8	0.9 ± 4.8	-	13.8 ± 1.8
Nea Kameni	3.4	-	Lava dome	5	11 M	61.7 ± 4.2	5.7 ± 1.2	9 ± 0.6	27.3 ± 1.5	-	-	0.2 ± 0.6	0.6 ± 1.5	-	-
					10 F	61.3 ± 2.8	4.4 ± 0.8	10 ± 0.3	10 ± 0.6	-	-	0.1 ± 0.3	0.2 ± 0.6	-	-

## Data Availability

Newly generated sequence data from this study are publicly available on the NCBI GenBank database, accession numbers OQ415536 to OQ415540.
